# Favorable Reoperation Rate at 2 Years Following Repair of Horizontal Cleavage Tears Using an All Suture-Based Technique: A Prospective, Multicenter Trial

**DOI:** 10.1016/j.asmr.2021.01.018

**Published:** 2021-03-13

**Authors:** Peter Kurzweil, Aaron J. Krych, Adam Anz, F. Winston Gwathmey, Gregory Loren, Matthew Lavery, David C. Flanigan

**Affiliations:** a Memorial Orthopaedic Surgical Group, Long Beach, California, U.S.A.; b Mayo Clinic, Rochester, Minnesota, U.S.A.; c Andrews Institute for Orthopaedics & Sports Medicine, Gulf Breeze, Florida, U.S.A.; d University of Virginia, Charlottesville, Virginia, U.S.A.; e CORE Orthopaedic Medical Center, Encinitas, California, U.S.A.; f OrthoIndy, Greenwood, Indiana, U.S.A.; g The Ohio State University Wexner Medical Center, Jameson Crane Sports Medicine Institute, Columbus, Ohio, U.S.A.

## Abstract

**Purpose:**

This prospective, multicenter trial evaluates the clinical success (as measured by reoperation rates and improvements in patient-reported outcome measures) of using circumferential compression stitches with all-suture techniques for horizontal cleavage tears (HCTs) of the meniscus.

**Methods:**

Investigators enrolled 30 patients (mean age, 38.2 years; standard deviation, 11.1 years) aged 18 to 60 years with HCTs in the symptomatic compartment at 8 centers in the United States who underwent HCT repair with all-suture circumferential stitches using a self-retrieving all-inside suture passing device. Postoperative follow-up visits were conducted at 6 months, 1 year, and 2 years. Study outcomes included freedom from reoperation of the index meniscus repair site; knee pain and function, as measured by International Knee Documentation Committee Knee evaluation (IKDC), Knee injury and Osteoarthritis Outcome Score, Lysholm Knee Scale, and Tegner Activity Scale; and serious complications observed during the study. Minimal clinically important difference at 1 year was assessed for IKDC and Lysholm.

**Results:**

Twenty-three patients had 2-year follow-up data available. Freedom from reoperation was 96.0% at 6 months (26/27, 91.7% at 1 year [23/25], and 82.6% at 2 years [19/23]). Significant improvement was observed in over baseline at 2 years for IKDC (36.7-82.5; *P* < .001), Knee injury and Osteoarthritis Outcome Score (52.2-89.3; *P* < .001), Lysholm (50.2-87.4; *P* < .001), and Tegner scores (3.3-5.3; *P* = .007). Minimal clinically important difference was met or exceeded for IKDC and Lysholm scores at 1 year 69.2% and 65.4% of patients, respectively. Four patients (6.7%) experienced serious complications, of which 2 were assessed as being related to the procedure.

**Conclusions:**

Repair of HCTs using all-suture circumferential stitches placed with a self-retrieving all-inside suture passing device leads to a favorable reoperation rate (17.4%), significant improvements in clinical outcomes, and an acceptable rate of serious complications (6.7%) at 2 years, supporting the viability of this treatment approach in this indication.

**Level of Evidence:**

Level IV, therapeutic case series.

Horizontal cleavage tears of the meniscus (HCTs) are situated parallel to the tibial plateau and divide the meniscus into inferior and superior portions.^1^ HCTs are a commonly encountered orthopaedic injury, by some estimates comprising up to one-third of meniscal tears,^2^ and can have both degenerative and traumatic origins.^3^ Compared with other common types of meniscal tears, HCTs have an increased incidence, number, and severity of chondral lesions.^3^

Unlike vertical-longitudinal tears, which have been deemed ideal targets for meniscal repair, HCTs have been traditionally treated either conservatively or with partial or total resection.^4^, ^5^, ^6^, ^7^, ^8^, ^9^, ^10^, ^11^ This treatment pathway has largely been determined based on concerns surrounding the technical difficulty of HCT repair, healing rates, and potential suture failure owning to mechanical stresses.^12^ However, data from the last decade have shifted clinical consensus by showing that repair can yield encouraging results. A 2014 systematic literature review of 9 studies (98 HCTs) reported an approximately 78% overall success rate following repair of HCTs.^12^ The various techniques that were used (open, inside-out, and all-inside repairs) produced comparable results. Second-look arthroscopy^13^ and follow-up via magnetic resonance imaging^14^ also have revealed full or partial healing following HCT repair.

In vitro biomechanical data indicate that circumferential stitching offers the greatest load to failure of all meniscus repair patterns.^15^ A self-retrieving all-inside suture repair device was created to enable surgeons to arthroscopically place circumferential sutures around HCTs, theoretically providing uniform compression of the tear edges.^16^ Cadaveric testing of HCT repair with circumferential compression stitches using this device concluded that it returned tibiofemoral contact pressures to near-normal.^17^

The purpose of this study was to evaluate the clinical success (as measured by reoperation rates and improvements in patient-reported outcome measures) of using circumferential compression stitches with all-suture techniques for HCTs of the meniscus. We hypothesized that the use of this technique would meet or exceed established success rates for HCT repair (e.g., 78%),^12^ and that patients would experience significant and sustained improvement in clinical function at 2 years.

## Methods

### Study Design

In this prospective, nonrandomized study, investigators enrolled otherwise-healthy patients aged 18 to 60 years with HCTs in the symptomatic compartment, confirmed by magnetic resonance imaging, at 8 centers in the United States from November 2014 (first patient enrollment) to November 2019 (last patient visit). Patients with Kellgren–Lawrence grade ≥3 osteoarthritis, body mass index ≥35, previous meniscal repair or meniscectomy of study meniscus, instability, malalignment of study knee >5° and/or requiring osteotomy and/or correction, expected to undergo any other primary treatment of the knee, pregnant or planning to become pregnant during the study, or a history of tobacco abuse (as determined by the principal investigator P.R.K.) were excluded. Reasons for removing a patient from the study following enrollment included, but were not limited to, the following: patient was uncooperative in adhering to the protocol requirements, including failure to participate in rehabilitation; investigator believed it was in the best interests of the patient; patient withdrew consent. All patients provided informed consent, in accordance with the governing institutional review board (local institutional review board or Western Institutional Review Board Protocol #20141243).

Consented subjects were included in the study only if, upon arthroscopic inspection, their meniscal study lesion met all of the following criteria established by the International Society of Arthroscopy, Knee Surgery and Orthopaedic Sports Medicine: (1) circumferential location of tear includes locations within 10 mm of the peripheral rim of the meniscus; (2) any location from anterior to posterior; (3) tear pattern was primarily horizontal or oblique in orientation (not to exceed 45 degrees from horizontal); (4) either lateral or medial compartment, but not both; (5) opposite compartment meniscal tear (if presented) limited to the central portion (i.e., zone 3/“white zone”); and (6) tear amenable to repair with all suture-based techniques.

Conversely, patients were excluded at this point if arthroscopy revealed their tear location and pattern did not meet these inclusion criteria; that their tear was intact or partially intact, and thereby did not require repair in the opinion of the investigator; that the meniscal tissue was of such poor quality that it would not hold a suture; that repair of any part of the meniscus required an implant other than a suture, or that a significant concomitant procedure was required on the study knee; or arthritis in the surgical knee Modified Outerbridge Grade III or greater.

There was no formal sample size calculation carried out for this clinical observational study, as it was not planned to test any formal hypothesis. Before the study commencement, it was planned to enroll 30 patients.

### Patient Population

**Fig 1:**
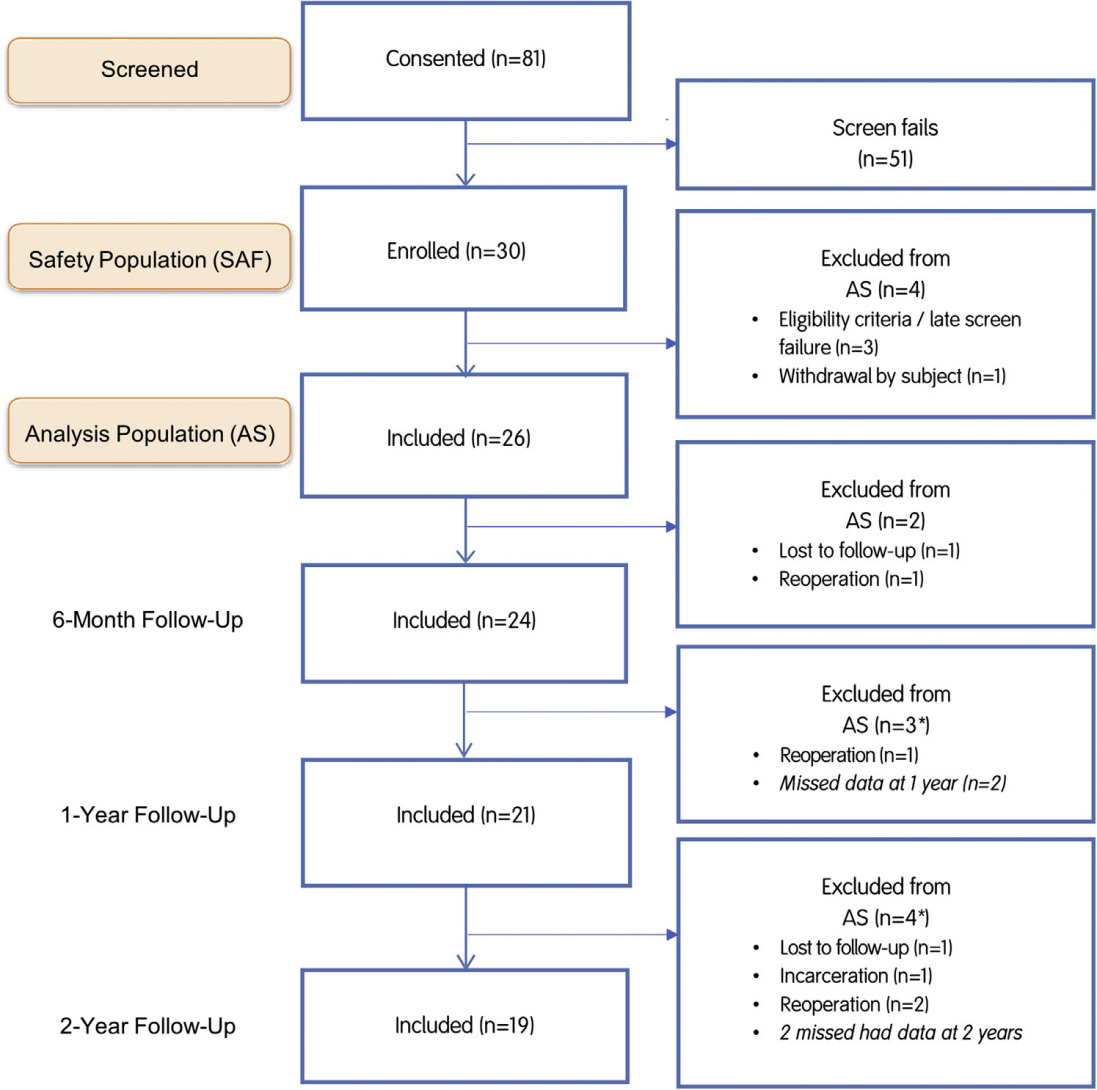
Patient flowchart.

Thirty patients were enrolled and underwent HCT repair with all-suture circumferential stitches using a self-retrieving all-inside suture passing device, forming the safety population. Four patients were excluded (late screen fail/did not meet inclusion criteria, n = 3; withdrawal by subject, n = 1), leaving 26 patients for the analysis population (Fig 1). Patient characteristics for both groups are provided in Table 1. Patients who were reoperated had outcomes data available until the latest follow-up period at which they were available.

**Table 1:** Patient Demographics for Both Safety and Analysis Populations

	Safety Population (n = 30)	Analysis Population (n = 26)
Demographics		
Mean age, SD	38.2 (11.1)	37.4 (11.6)
Mean BMI, SD	25.7 (3.5)	25.7 (3.6)
Male/female	20/10	17/9
Mean duration of symptoms before surgery, wk, SD	74.1 (136.1)	75.8 (144.1)
Mean time since injury, wk, SD	27.4 (24.8)	22.2 (21.8)
Tear pathology		
Side of tear		
Left knee/right knee	18/12	17/9
Cause of tear		
Degenerative	15	12
Traumatic	11	10
Undetermined	4	4
Meniscus compartment		
Medial	19	16
Lateral	8	8
Missing data	3	2
Tear depth type		
Partial	16	15
Complete	11	9
Missing data	3	2
Primary orientation		
Horizontal	28	24
Oblique	2	2
Length of tear, mm, SD	17.7 (5.9)	18.4 (5.8)
Rim with deepest zone		
Zone 1	14	11
Zone 2	13	13
Zone 3	3	2
Surgical details		
Mean surgical time, min, SD	63.2 (19.6)	64.2 (20.3)
Mean meniscal repair time, min, SD	35.6 (11.3)	36.2 (10.0)
Mean number of sutures used, SD	4.9 (2.7)	5.1 (2.8)
Surgical technique		
All inside	19	16
All-inside/inside-out	2	2
All-inside/outside-in	1	1
Outside-In	2	2
Outside-in/all inside	3	3
Outside-in/inside-out	1	1
Other	1	1
Missing	1	0

BMI, body mass index; SD, standard deviation.

### Study Procedure

All patients received all-suture circumferential stitches using a self-retrieving all-inside suture passing device (NovoStitch PRO Meniscal Repair System; Smith & Nephew, Andover, MA), with inside-out or outside-in hybrid repairs. The device passes size 2-0 braided, nonabsorbable, surgical suture through soft tissue in arthroscopy surgery and comprises a handheld surgical instrument to which cartridges preloaded with suture are attached. The recommended spacing of sutures was 5 mm. Patients followed the investigators’ preferred rehabilitation plan based on the particular tear and repair characteristics.

### Study Outcomes

Patients were assessed immediately postoperatively, and thereafter at 6 months, 1 year, and 2 years. Study outcomes included freedom from reoperation of the index meniscus repair site (primary end point, calculated from the safety population who had a known outcome at that time point), and improvements in knee pain and function, as measured by International Knee Documentation Committee (IKDC) Knee evaluation, Knee injury and Osteoarthritis Outcome Score, Lysholm Knee Scale, and Tegner Activity Scale. Meniscus healing was assessed in a subset of available patients who volunteered to undergo second-look in-office needle arthroscopy (e.g., VisionScope) at 6 months. Complete healing was defined by the surgeon upon visual inspection of the meniscus. A safety analysis was conducted, with serious complications reported throughout the study described herein.

### Statistical Analysis

Minimal clinically important difference (MCID) was established for IKDC and Lysholm as 16.7 and 10.1, respectively, at 1 year.^18^ Appropriate descriptive statistics were used for baseline characteristics, procedural variables, reoperation rate of the index meniscal repair site, and major complications. Unless otherwise stated, all significance tests were 2-sided, performed at the 5% significance level. Resulting *P* values were quoted and 95% 2-sided confidence intervals (CIs) were generated where appropriate. All analyses were performed in SAS 9.4 (SAS Institute, Cary, NC) or later. As appropriate, a repeated-measure analysis of variance or Wilcoxon signed rank test was completed for each outcome measure. A *P* value ≤0.05 was considered significant.

## Results

### Efficacy Outcomes

Freedom from reoperation using the safety population for which we had an outcome at that time point was 96.0% at 6 months (26/27; 95% CI 81.0-99.9), 91.7% at 1 year (23/25; 95% CI 76.5-99.1), and 82.6% at 2 years (19/23; 95% CI 69.3-96.2). Of 23 patients for whom relevant 2-year efficacy outcome data were available, 4 (17.4%) required reoperation during the study (95% CI 5.0-38.8). Mean time to reoperation was 43.2 (SD ±31.9) weeks from the available patient data. Two subjects had meniscectomy/partial meniscectomy of the white zone at reoperation. The other 2 subjects did not undergo meniscus repair.

**Fig 2:**
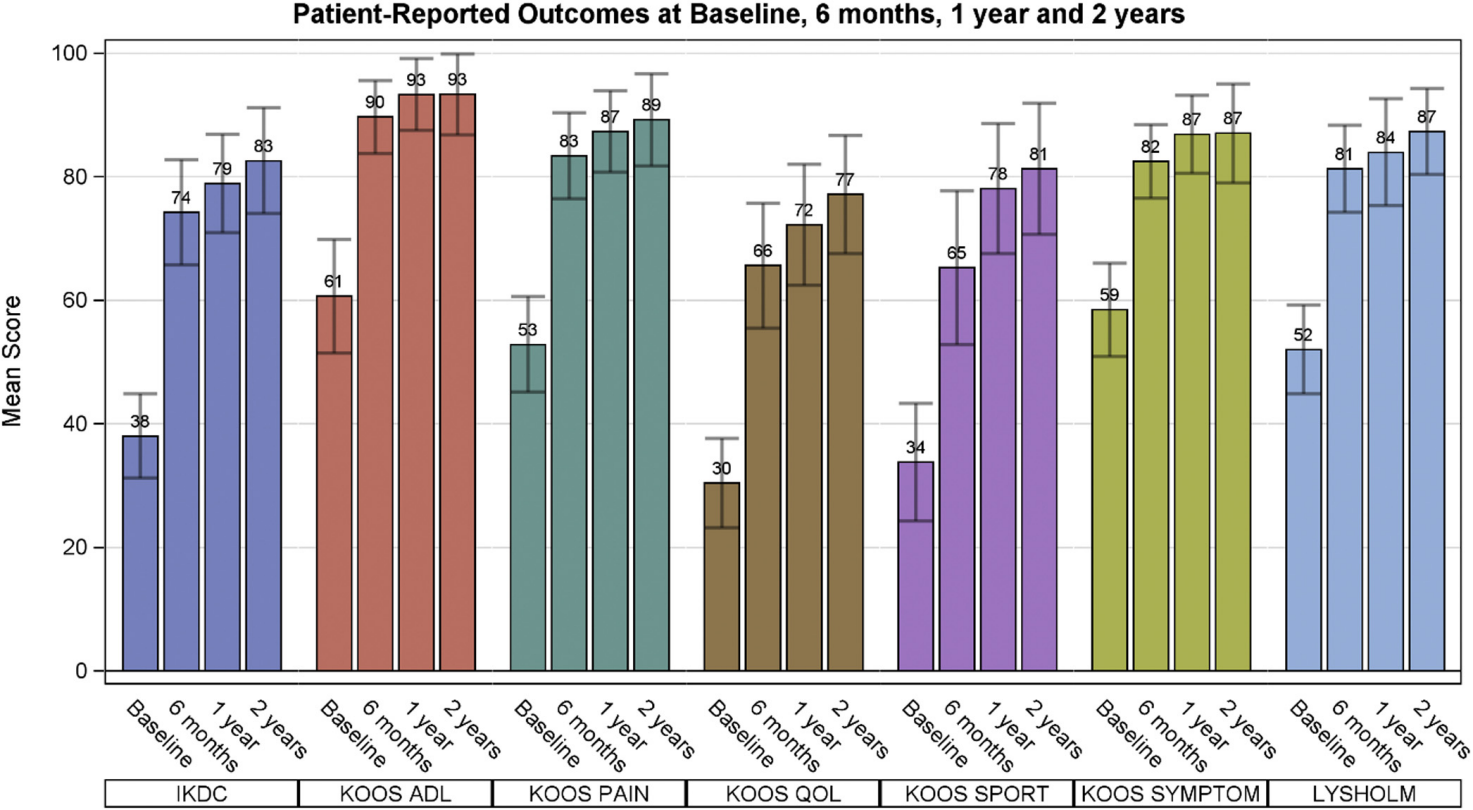
Patient-reported outcome measures (mean ± 95% confidence interval) at baseline, 6 months, 1 year, and 2 years. A Wilcoxon signed rank test pairwise comparison of baseline to each visit revealed a *P* value of <.001 for all outcome measures. (ADL, activities of daily living; IKDC, International Knee Documentation Committee Knee evaluation; KOOS, Knee injury and Osteoarthritis Outcome Score; QOL, quality of life.)

All patient-reported outcomes showed significant improvements (*P* < .05) over baseline at the initial 6-month follow up, and these improvements were maintained at each subsequent follow-up points (Fig 2). The MCID for improvement in IKDC and Lysholm scores at 1 year was met by 69.2% (95% CI: 48.2-85.7) and 65.4% (95% CI: 44.3-82.8) of patients, respectively. The mean (standard deviation) Tegner Activity Scale scores increased from baseline (3.3 ± 2.4) to subsequent follow-up visits at 6 months, 1 year, and 2 years (4.6 ± 2.4, *P* = .039; 5.1 ± 2.2, *P* = .008; 5.3 ± 2, *P* = .007; respectively).

Of the 11 patients in the analysis population who were visually assessed via second-look needle arthroscopy at 6-month follow up, all showed that complete healing of the tear.

### Safety Outcomes

There were 30 patients in the safety population, which included all enrolled patients who received the protocol-defined treatment of suture-based meniscal repair (including 4 patients with late screen fails/withdrawals). Four patients (13.3%) experienced serious complications. For 2 of these patients, the serious complications were not assessed as being related to the device and/or procedure. One patient experienced further, nonrepairable tear of the meniscus, which was resolved with a subsequent meniscectomy. The other patient experienced pain in the right (contralateral) knee, which was treated and resolved with partial medial meniscectomy and medial compartment chondroplasty.

Two patients experienced anticipated serious adverse effects assessed as being unrelated to the study device but related to the procedure. One patient experienced meniscus repair that did not heal and recurrent symptoms of meniscus tear, which were resolved with a partial meniscectomy. One patient reported hot and cold sweats, fever and swelling and pain of the knee, and was found to have elevated inflammatory markers but no leukocytosis following an incident where he slipped and put weight on the operative knee. Surgery was performed to mitigate this though the outcome was not marked as resolved at the time of reporting.

There were no unanticipated serious adverse device effects, device deficiencies, or deaths reported during the study.

## Discussion

At 2 years’ follow-up, patients undergoing circumferential meniscus repair of HCTs experienced a favorable 17.4% reoperation rate. Meaningful improvements in knee pain and function following suture-based repair of the horizontal meniscal tear were observed from patient-reported outcome measures, with statistically significant differences at all follow-up visits.

These results support the concept that HCTs can be successfully operated with circumferential compression stitches using an all-suture device. These data join a relatively small but increasing number of clinical studies supporting the repair of HCTs. This is a notable shift from the preferred management approaches to HCTs in years prior, which recommended either conservative approaches or meniscectomy, despite the often-unfavorable outcomes obtained.

The decision to leave HCTs untreated or to manage them nonoperatively via relatively conservative interventions (e.g., physical therapy, medications, and/or injections) can have deleterious clinical ramifications. Relative to other meniscal tear types, HCTs have an increased incidence and severity of chondral lesions.^3^ HCTs can significantly reduce contact area and increase tibiofemoral contact pressure, thereby further escalating the risk of subsequent cartilage degeneration.^17^^,^^19^ Widening and deformation of existing HCTs occurs during knee flexion, with worsening clinical symptoms observed as the tear size increases, acting as a potential contributor to patient pain.^20^

In the published literature, meniscectomy remains the most commonly described surgical intervention for HCTs. In a 2020 systematic literature review of studies into surgical treatment of HCTs, Shanmugaraj et al.^21^ reported that that majority of knees underwent meniscectomy instead of repair (67.2% vs 32.8%). However, these numbers may reflect historical surgeon preference rather than contemporary clinical practice, as meniscectomy has fallen out of favor in the past decade after well-designed studies indicated it did not lead to superior 1-year outcomes when compared with conservative interventions or sham surgery (a simulation of standard arthroscopic partial meniscectomy).^22^^,^^23^ Furthermore, resection increases the risk of late osteoarthritis compared with meniscal repair.^24^ HCTs are a significant risk factor for progression to high-grade osteoarthritis within 5 years after partial meniscectomy.^25^^,^^26^ Resection of both leaflets results in decreased contact area and increases contact pressure, which can lead to onset of osteoarthritis development.^27^ Recent evidence indicates that repair reduces the relative risk of osteoarthritis almost 3-fold relative to meniscectomy.^28^

If the relative limitations of conservative management and meniscectomy for HCTs were apparent, there was still a prevailing belief that repair HCTs had inferior healing compared with other meniscal repair types. This notion was countered by a 2014 systematic literature review by Kurzweil et al.^12^ In reviewing 9 publications (98 HCTs), the authors reported that HCT repair had an overall success rate of 78%, comparable with the rates observed with repair of other meniscal tear types.

A recently published systematic literature review by Morris et al.^29^ reaffirmed that HCT repair retains its relative success to other tear type repairs. After including studies published in the intervening years, the authors analyzed 19 studies (289 knees) of HCT repair and reported an 11.7% risk of reoperation.

In the current study, we report a freedom from reoperation rate of 96.0% at 6 months, 91.7% at 1 year, and 82.6% at 2 years. Neither Kurzweil et al. or Morris et al. provide mean follow-up times to contextualize their rates of success or failure, which prevents us from comparing our rates with theirs at specific follow-up points.^12^^,^^29^ Nonetheless, the 2-year success rate is in line with positive expectations set by those systematic literature reviews and provides additional evidence that surgical repair is a viable treatment option in HCTs.

Our study also benefits from its prospective design, which to date has been lacking in the literature of HCT repair. Of 19 HCT repair studies identified by Morris et al.,^29^ there were only 2 prospective cohort studies and one randomized controlled trial.

Shanmugaraj et al.^21^ assessed clinical outcomes for available studies of HCT repair. The mean postoperative outcomes for the IKDC, Lysholm, and Tegner scores provided in their overview at mean 30.6 ± 11.1 months were comparable with those observed at 24-month follow up in the current analysis (86.7 vs 82.5, 91.3 vs 87.4, and 6.6 vs 5.3, respectively). As in their overview, the MCID was achieved for improvement in IKDC and Lysholm scores in the majority of patients our series as well. The various Knee injury and Osteoarthritis Outcome Score subscales analyzed in our study also indicate the effective and durable clinical outcomes that can be obtained with HCT operated with circumferential compression stitches using an all-suture device. Our analysis population was a mean of 37.4 years old, and younger patients are thought to experience superior functional results following repair.^30^ Therefore, these results may reflect the advantages of this population.

Among 30 patients, 2 (6.7%) experienced serious complications assessed as being related to the study procedure. Shanmugaraj et al. reported a greater rate of complications following HCT repair when compared with partial meniscectomy (12.9% vs 1.3%, respectively).^21^ In their recent systematic literature review of HCTs undergoing repair, Morris et al.^29^ reported an overall complication rate of 20.3%, though they noted that this fell to a more modest rate of 5.7% when excluding failure of the repair. However, there are limitations when comparing the rates of adverse events to other studies.^21^^,^^29^ Studies may classify adverse events or complications using different criteria, and may not clarify whether they are related or unrelated to the device or procedure. The way that adverse events or complications are reported can vary between studies and sites (in multicenter studies), and reoperations are not always reported as adverse events. Moreover, the small sample size of the current study may further hamper direct comparison with other studies and systematic reviews.^21^^,^^29^

The arthroscopic repair of HCTs with circumferential stitching is proposed to offer superior load to failure^15^ and near-normal restoration of tibiofemoral contact pressures.^17^ It also affords the operating surgeon additional advantages. It can be performed with an arthroscopic technique, which is advantageous as open repair has been reported to have a greater failure rate in HCTs.^21^ The ease of placement of arthroscopic all-inside circumferential sutures for posterior portions of meniscus limited the need for extensive inside-out or outside-in exposure (and risks). Circumferential compression sutures are an alternative to conventional fixators, which may not provide satisfactory coaptation and stable fixation for certain tear types. A major advantage of the circumferential compression sutures delivered with the NovoStitch PRO Meniscal Repair System used in this study is the ability of the surgeon to repair multiple tear types, with a greater degree of versatility than previously thought possible.

### Limitations

This study is limited by its relatively small sample size (30 patients in safety population, 26 in analysis population). From an eligible population of 30 patients, 19 had available patient-reported outcome measures at 2-year follow-up, potentially limiting the power of the clinical assessment. In addition, only 11 patients in the analysis population underwent in-office arthroscopy. In-office needle arthroscopy diagnostic testing is associated with only minimal risks, and provides an invaluable means for assessing the state of early meniscal healing following HCT repair. However, patients understandably may be unwilling to undergo an additional invasive procedure relatively soon following their index operation. There was no power calculation conducted to determine a sample size for this study, as it was not planned to test any formal hypothesis. The lack of a comparator arm means we could not analyze how these results differ with other types of all-inside meniscus repair, meniscectomy, or non-operative interventions. Finally, the study protocol did not establish specific criteria for reoperation. As this was a subjective decision made by the investigators, there is the potential for bias.

## Conclusions

Repair of HCTs using all-suture circumferential stitches placed with a self-retrieving all-inside suture passing device leads to a favorable reoperation rate (17.4%), significant improvements in clinical outcomes, and an acceptable rate of serious complications (6.7%) at 2 years, supporting the viability of this treatment approach in this indication.

## Supplementary Data

ICMJE author disclosure forms
